# High-throughput phenotyping of plant resistance to aphids by automated video tracking

**DOI:** 10.1186/s13007-015-0044-z

**Published:** 2015-01-30

**Authors:** Karen J Kloth, Cindy JM ten Broeke, Manus PM Thoen, Marianne Hanhart-van den Brink, Gerrie L Wiegers, Olga E Krips, Lucas PJJ Noldus, Marcel Dicke, Maarten A Jongsma

**Affiliations:** Laboratory of Entomology, Wageningen University, P.O. Box 16, 6700 AA Wageningen, The Netherlands; Laboratory of Plant Physiology, Wageningen University, P.O. Box 16, 6700 AA Wageningen, The Netherlands; Plant Research International, Wageningen University and Research Center, P.O. Box 16, 6700 AA Wageningen, The Netherlands; Noldus Information Technology bv, P.O. Box 268, 6700 AG Wageningen, The Netherlands

**Keywords:** Aphids, Arabidopsis, Automated video tracking, Host plant resistance, *Lactuca sativa*, Phenotyping, Piercing-sucking insects, *Arabidopsis thaliana*

## Abstract

**Background:**

Piercing-sucking insects are major vectors of plant viruses causing significant yield losses in crops. Functional genomics of plant resistance to these insects would greatly benefit from the availability of high-throughput, quantitative phenotyping methods.

**Results:**

We have developed an automated video tracking platform that quantifies aphid feeding behaviour on leaf discs to assess the level of plant resistance. Through the analysis of aphid movement, the start and duration of plant penetrations by aphids were estimated. As a case study, video tracking confirmed the near-complete resistance of lettuce cultivar ‘Corbana’ against *Nasonovia ribisnigri* (Mosely), biotype Nr:0, and revealed quantitative resistance in Arabidopsis accession Co-2 against *Myzus persicae* (Sulzer). The video tracking platform was benchmarked against Electrical Penetration Graph (EPG) recordings and aphid population development assays. The use of leaf discs instead of intact plants reduced the intensity of the resistance effect in video tracking, but sufficiently replicated experiments resulted in similar conclusions as EPG recordings and aphid population assays. One video tracking platform could screen 100 samples in parallel.

**Conclusions:**

Automated video tracking can be used to screen large plant populations for resistance to aphids and other piercing-sucking insects.

**Electronic supplementary material:**

The online version of this article (doi:10.1186/s13007-015-0044-z) contains supplementary material, which is available to authorized users.

## Background

More than 100 aphid species (Aphididae) are economically significant pest insects and most crops are host to at least one species [1]. Aphids feed on phloem sap, and to reach the phloem they move their stylets between plant cells towards a sieve element, making short punctures in cells along the way. Most probes are prematurely interrupted in the epidermis and mesophyll. When, however, a phloem vessel is reached, aphids can ingest phloem sap continuously for many hours or even days [2]. Although aphids inflict little tissue damage, they transmit plant viruses and deplete host plants of photoassimilates and free amino acids [3,4]. In wild plant populations aphids rarely constitute pests due to effective natural defence strategies, such as epicuticular waxes, protease inhibitors, and induced production of secondary metabolites [4-12]. After generations of domestication many of these defence traits have been diminished or lost in cultivated plants, making them vulnerable targets of herbivorous insects [13,14]. The genetic backgrounds of resistance mechanisms still remain largely elusive and genomics studies strongly depend on the capacity for phenotyping large panels of plants. Few high-throughput methods have been established for assessing plant resistance to insect herbivores, such as aphids or other piercing-sucking insects [15-20]. Generally, two approaches are used to quantify the level of plant defence against aphids; either assessment of aphid population development or investigation of aphid feeding behaviour. Aphid population assays are generally the most demanding in terms of time and space, since they require the availability of a climate-controlled compartment for 1 or 2 weeks and extensive manual work [21-23]. On the contrary, aphid feeding behaviour can be measured within a couple of hours via the Electrical Penetration Graph (EPG) technique. EPG recording delivers electrical waveforms comprising information on the plant tissue that is penetrated (phloem vessel, xylem vessel or other cells) and the stylet penetration activity (cell puncture, salivation, ingestion, penetration difficulties) [24,25]. EPG studies have shown that aphids prolong phloem ingestion on suitable host plants and delay and reduce feeding on resistant or non-host plants [26-33]. The high specificity of the information about plant tissue and key components of aphid behaviour, makes this methodology appealing for exploring defence mechanisms. A drawback of EPG is, however, the restricted capacity, generally 8 plants per setup [34], and the labour-intensive nature of wiring aphids and annotating electrical signals.

Here, we present the methodology and validation of image-based tracking of aphid feeding behaviour. Automated video tracking was introduced in the early 1990s and has since been used in many animal behaviour studies [35-41]. Video tracking involves software-engineered pattern analysis of grids of pixels in order to quantify the location and movement of subjects over time. In this study, we used movement patterns of the central body point of aphids to estimate the duration of plant penetrations made by the aphid’s mouth parts. Previous EPG studies showed that probes shorter than approximately 3 minutes represent penetrations in the epidermis and/or mesophyll [26], and that probes involving phloem uptake last on average at least 25 min [27,42,43]. This allowed us to discriminate test probes from putative phloem uptake events in video observations in order to identify plants that are resistant to aphids. We benchmarked the performance of the high-throughput video tracking platform against EPG recordings and aphid population development assays, using natural accessions of *Arabidopsis thaliana*, and lettuce cultivars, *Lactuca sativa*, in combination with the green peach aphid, *Myzus persicae* (Sulzer), and the black-currant lettuce aphid, *Nasonovia ribisnigri* (Mosely) (Hemiptera: Aphididae), respectively.

## Results

### Tracking aphid feeding behaviour

**Figure 1:**
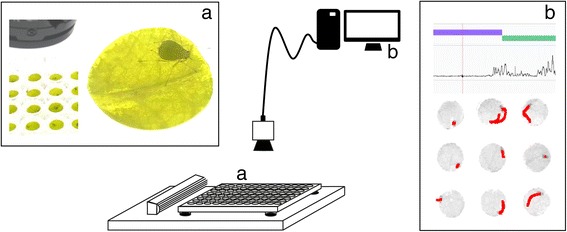
**Video tracking platform.** A stationary camera is mounted above a microtitre plate which is placed on top of a backlight unit with ventilation at the left. Wells in the microtitre plate contained a leaf disc and an aphid **(a)**. Cling film was wrapped around the plate to prevent aphids from escaping. The camera was connected to a computer with EthoVision® XT video tracking software **(b)**. Movements of the aphid’s centre point were automatically tracked (red track shows movements across 30 seconds). With this information the software calculated aphid velocity (line graph) and estimated probing (purple bar) and non-probing events (green bar).

**Figure 2:**
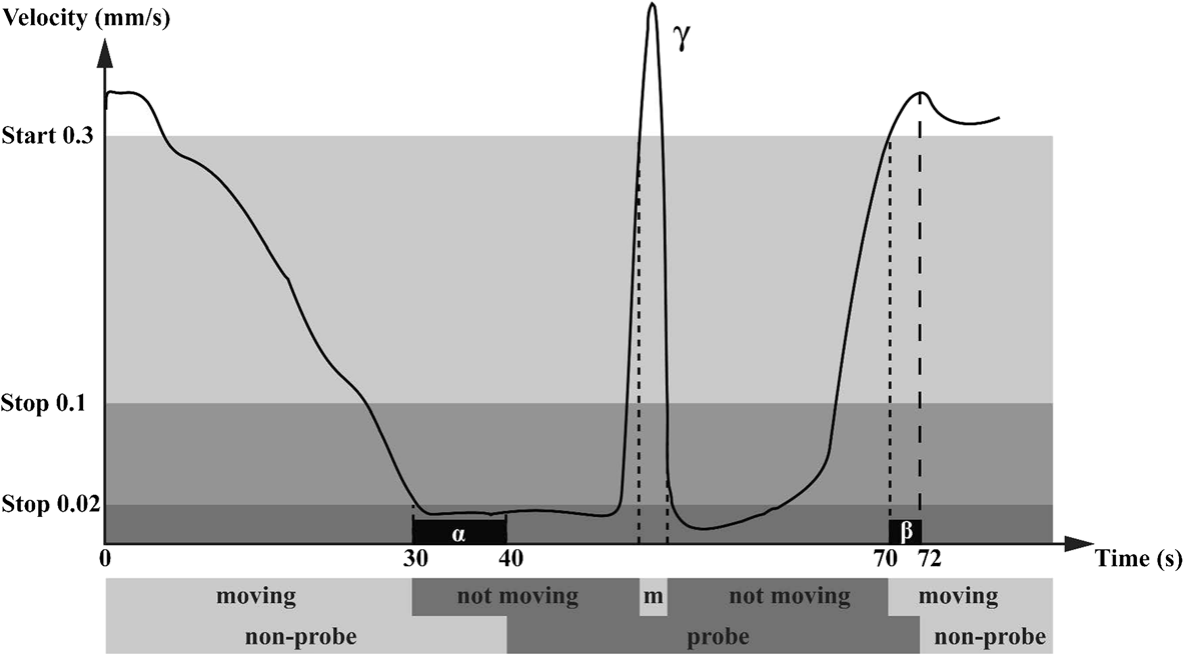
**Velocity thresholds for registration of probes.** An example of how aphid feeding behaviour was measured using a resolution of 275 pixels per mm^2^. Subject states can be defined as ‘moving’ or ‘not moving’ by means of two thresholds: the start velocity at which the subject begins to move, and the stop velocity at which the state changes from moving to not moving. Probe starts were recorded if the velocity of the aphid’s centre point dropped below 0.02 mm/s for at least 10 seconds (α). Probe stops were recorded if the velocity of *M. persicae* aphids exceeded 0. 3 mm/s for at least 2 seconds (β), or 0.35 mm/s for at least 2 seconds in the case of winged *N. ribisnigri* aphids. To avoid premature probe endings due to short movements during probing (event γ), probe stops were only recorded when velocity increased above 0. 1 mm/s for more than 2 seconds. Figure adjusted from the EthoVision XT Reference Manual (version 8) [44].

Automated video tracking of aphid feeding behaviour was performed using video tracking software and a stationary camera mounted above 20 no-choice arenas. We introduced one aphid onto each arena, consisting of an agar substrate almost completely covered by a leaf disc, and recorded 20 arenas simultaneously with a frame rate of 25 frames s^−1^ (Figure 1, Additional file 1: Figure S1). Because the aphid’s mouthparts were not visible in the multi-arena setup, we made the assumption that when the aphid’s centre point was located on the leaf disc and did not move, the aphid was penetrating the leaf tissue with its stylets. By assessing video images by eye, we defined velocity thresholds for the start and end of probing events of two aphid species, *M. persicae* and *N. ribisnigri* (Figure 2, Additional file 1: Figure S2). According to our observations, the software was more vulnerable to premature probe endings of *N. ribisnigri* due to body movements during probing (such as event γ in Figure 2). As this aphid species is somewhat larger (±1.9 mm body length, versus ± 1.7 mm for *M. persicae*), movements around the fixated mouth resulted in a higher tangential velocity, and therefore required a higher velocity threshold.

### Accuracy

**Figure 3:**
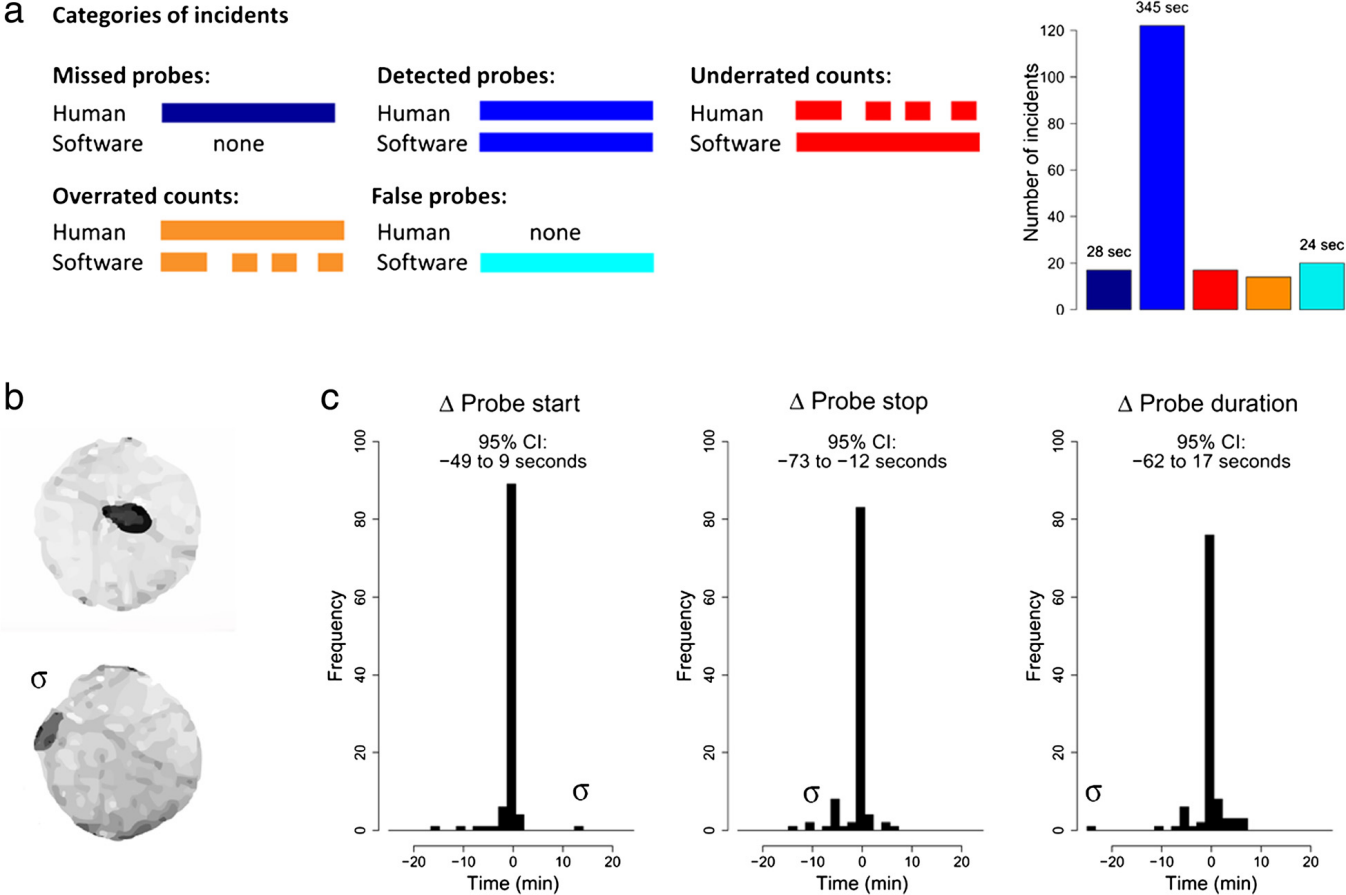
**Accuracy of automated tracking in comparison to human observations.** *M. persicae* feeding behaviour was measured on Arabidopsis leaf discs by automated video tracking and human observations simultaneously. **(a)** Out of 139 probes of 16 aphids scored by hand, 88% was detected by automated video tracking. Probes were considered a match when their duration overlapped at least partially. Some of the detected probes were matched by too few (underrated) or too many (overrated) probes. For these situations, the amount of missed or redundant probes is shown. 17 Probes went undetected and 20 false probes were recorded. Mean duration per probe is shown above the bars. **(b)** Screenshots of the top-view video used for automated tracking. The lower image (σ) shows an aphid positioned on the edge of the leaf disc for more than 20 min, causing overrated probe counts by the software due to continuous switching between an “on the leaf disc” and “off the leaf disc” status. **(c)** Differences between software and human observations per matched probe. 95% Confidence Intervals are shown above the histograms. Negative values correspond to too early probe starts, too early probe endings, resp. too short duration of probes compared to the human observations. In case of overrated probe counts, the probe with the most similar duration as the manually scored probe was included. The outlier caused by the example in **(b)** is annotated with σ.

**Figure 4:**
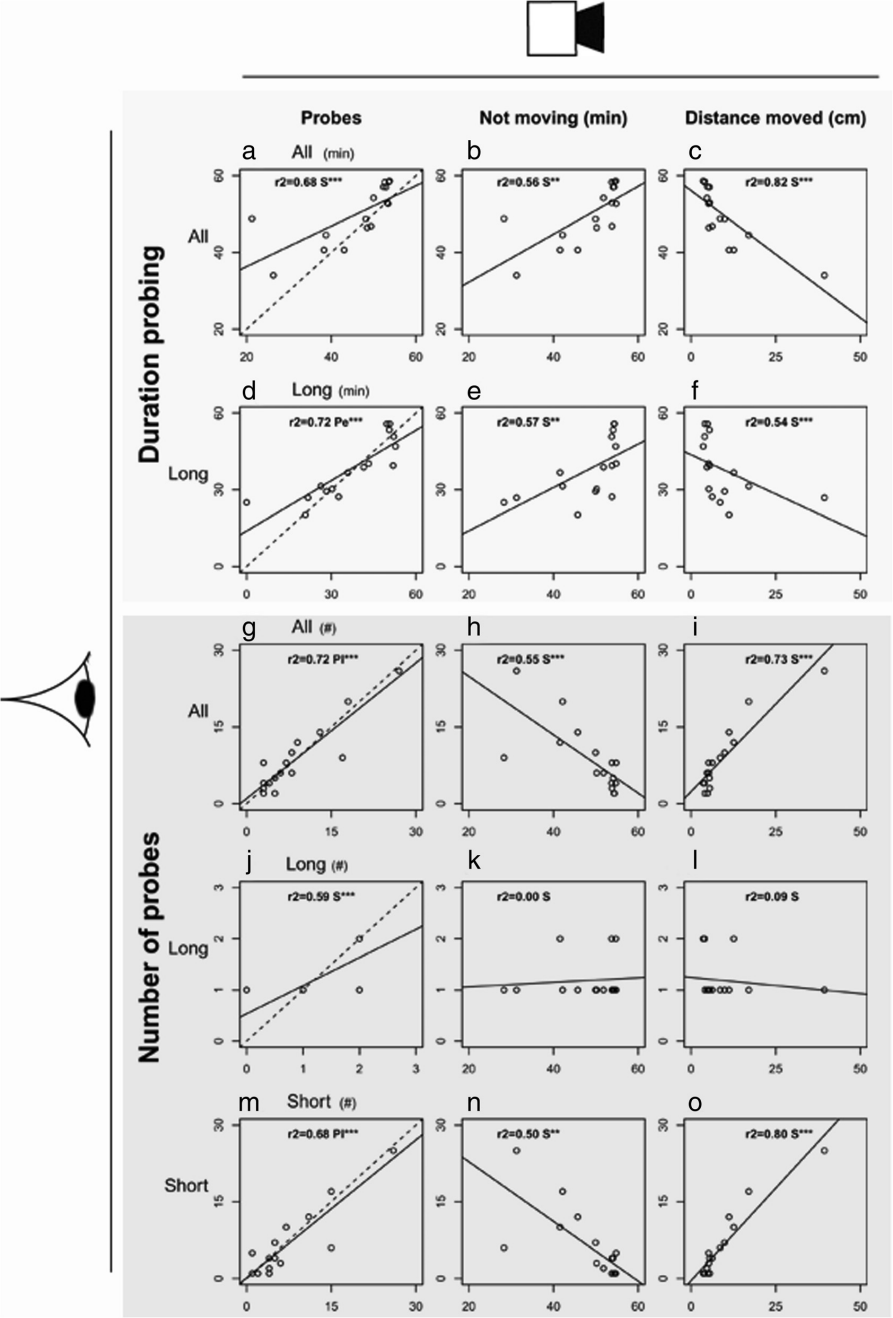
**Correlation between automated video tracking and human observations.** *M. persicae* behaviour was measured by automated video tracking (x-axes) and human observations simultaneously (y-axes). Three categories of probes were distinguished: All probes, Long probes (>15 min), and Short probes (<3 min). The duration (min) and number of probes measured by human observations were compared to: **(a,d,g,j,m)** the duration (min) and number of probes (all, long, and short probes) measured by video tracking, **(b,e,h,k,n)** the total time not moving (min), and **(c,f,i,l,o)** the distance moved by the aphids (cm) (*P < 0.05; **P < 0.01; ***P < 0.001, Pe = Pearson correlation test, Pl = Pearson correlation test on log transformed data, S = Spearman correlation test, dashed lines represent a hypothetical r^2^ = 1, n = 16 recordings of 1 aphid for 55 min, 275 pixels per mm^2^).

To test the accuracy of the platform, we performed automated video tracking and human observations simultaneously. A camera was attached to a stereo microscope to deliver a side-view on the arena for manual scoring of probes (Additional file 2). Among a total of 139 probes of 16 different *M. persicae* aphids scored by hand, 88% was detected with video tracking (Figure 3a). Undetected and false positive probes involved only short events (<3 min). Of the detected probes, 19% was either underrated (multiple ‘true’ probes were considered as one probe), or overrated (one ‘true’ probe was translated into multiple probes by the software). Underrated samples were caused by undetected probe stops due to slow movements below the velocity threshold. Overrated samples were caused by false probe stops when, for example, the aphid was immobile on the edge of the leaf disc and the assigned position continuously switched between an “on the leaf disc” and “off the leaf disc” status (Figure 3b). Three times this incident occurred, leading to 17 redundant probes of which 10 were filtered out automatically (see Methods, section Software settings). Other reasons for premature probe stops were abdominal movements during probing related to e.g. reproduction or honeydew excretion. The longer probes lasted, the higher the risk was of encountering such incidents. Indeed automatically tracked probes were in general biased to end 73 to 12 seconds too early (Figure 3c), and the total duration of probing was underestimated (on average 46 min ± 2.5 min standard error, versus 50 min ± 1.9, P = 0.01, Mann–Whitney *U* test, total observation duration: 55 min). Nevertheless, the recorded number and duration of probes were highly correlated to human observations (Figure 4, average r^2^ = 0.7 with 275 pixels per mm^2^). Other parameters, such as distance moved, were also highly correlated with feeding behaviour in general, but were less informative with regard to long probes (Figure 4l). Although automated video tracking did not achieve a precision as high as manual scoring, it enabled observing multiple arenas simultaneously. In the above described tests, we used 275 pixels per mm^2^, equal to a coverage of 20 arenas with our 768 × 576 pixels camera. To determine whether the capacity could be increased, we repeated the experiment with only 155 pixels per mm^2^, equal to a coverage of 35 arenas, but found that reduced resolution resulted in decreased correlations with human observations (average r^2^ < 0.5).

### Benchmarking against EPG recording with Arabidopsis

**Figure 5:**
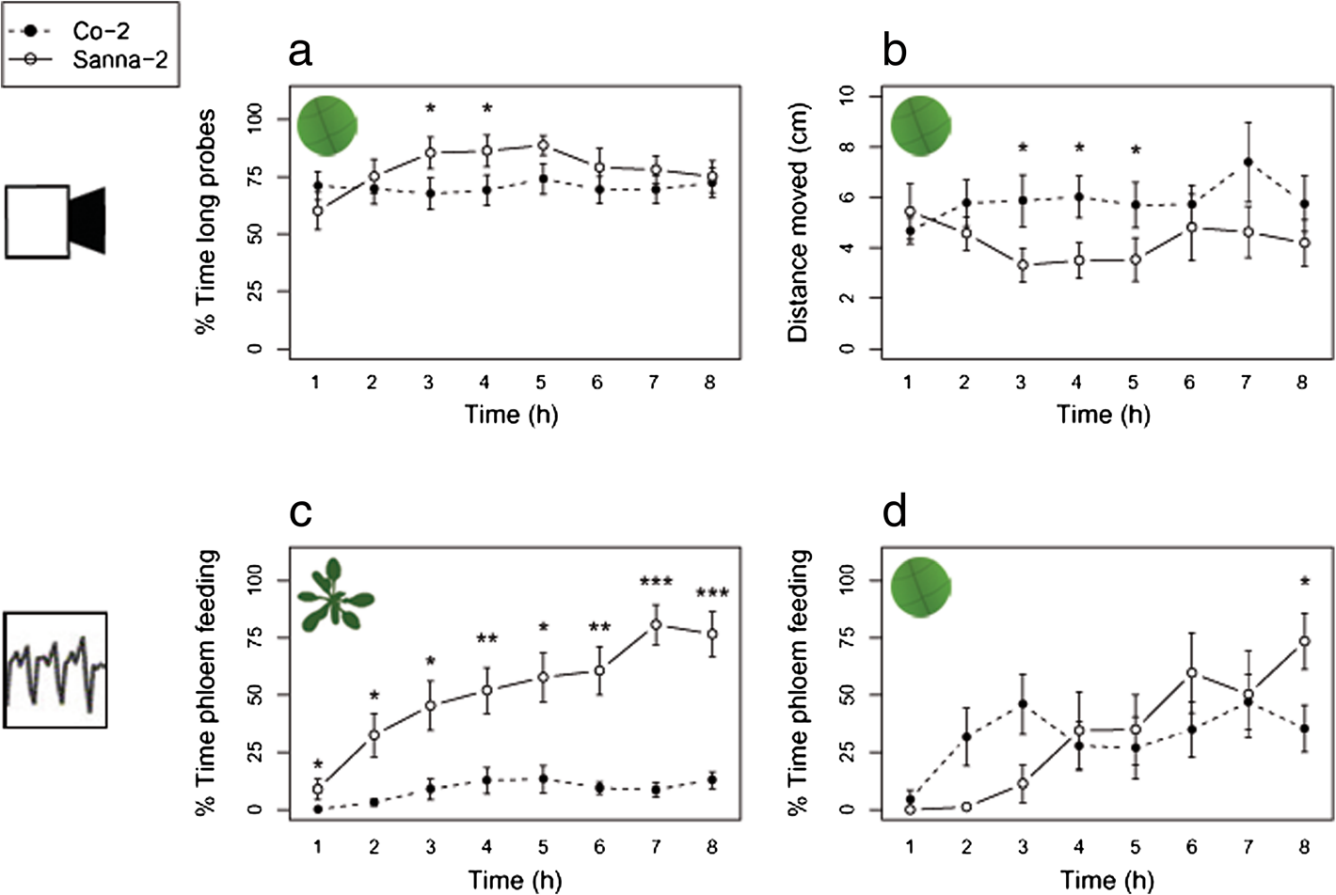
**Behavioural parameters of** ***M. persicae*** **on two natural Arabidopsis accessions, Co-2 (resistant) and Sanna-2 (susceptible). (a)** Percentage of the time spent on long probes (>25 min), and **(b)** distance moved (cm) were measured by automated video tracking. Percentage of the time spent on phloem feeding (waveform 5) were measured by **(c)** EPGs on intact plants, and **(d)** EPGs on leaf discs (Mann–Whitney U test, *P < 0.05; **P < 0.01; ***P < 0.001, video tracking: Co-2 n = 20, Sanna-2 n = 17, EPG recording intact plants: n = 19, EPG recording leaf discs: Co-2 n = 9, Sanna-2 n = 8, error bars represent standard error).

**Figure 6:**
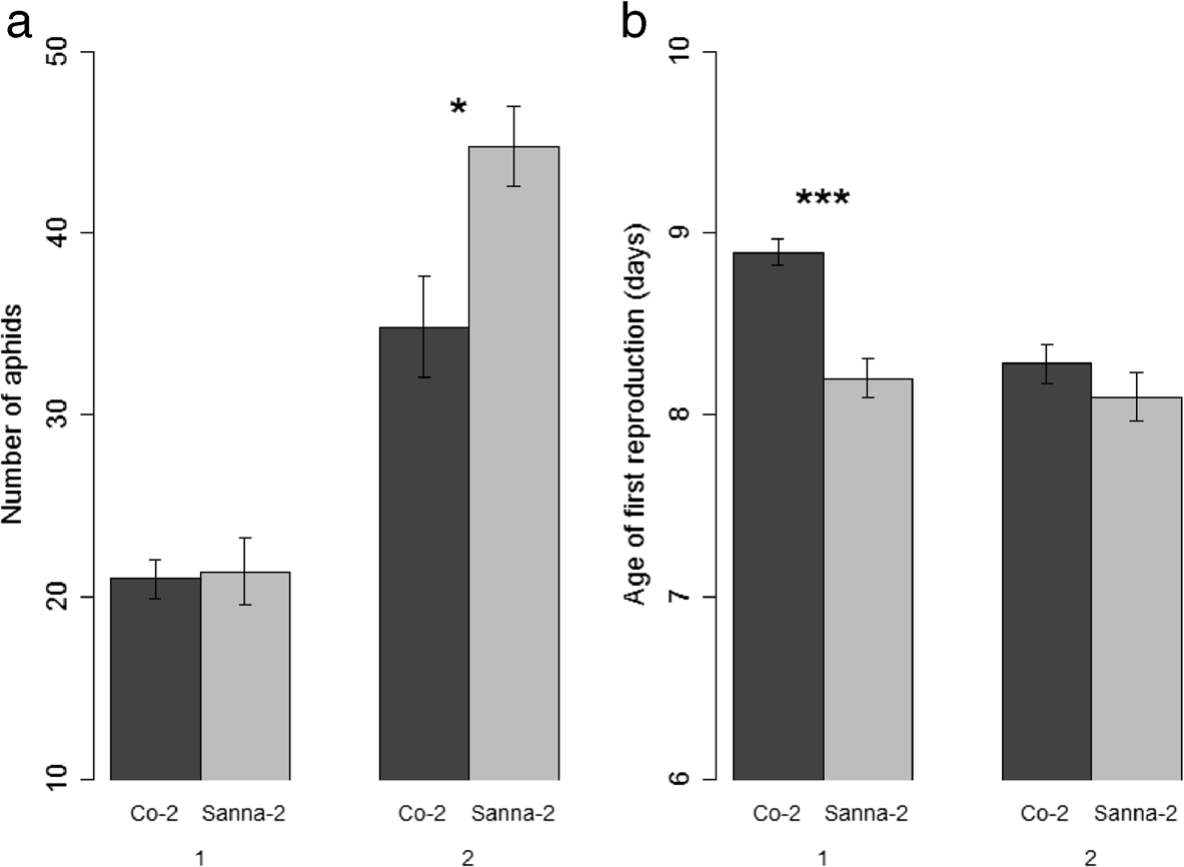
**Reproduction of** ***M. persicae*** **on two Arabidopsis accessions.** One neonate aphid was introduced to a 2.5-week-old plant (assay 1) or a 3.5-week-old plant (assay 2). **(a)** Total number of aphids per plant 2 weeks after infestation. **(b)** Days until the first nymph was produced by the aphid (Mann–Whitney U test, *P < 0.05, ***P < 0.001, assay 1: Co-2 n = 19, Sanna-2 n = 15, assay 2: Co-2 n = 14, Sanna-2 n = 13, error bars represent standard error).

**Figure 7:**
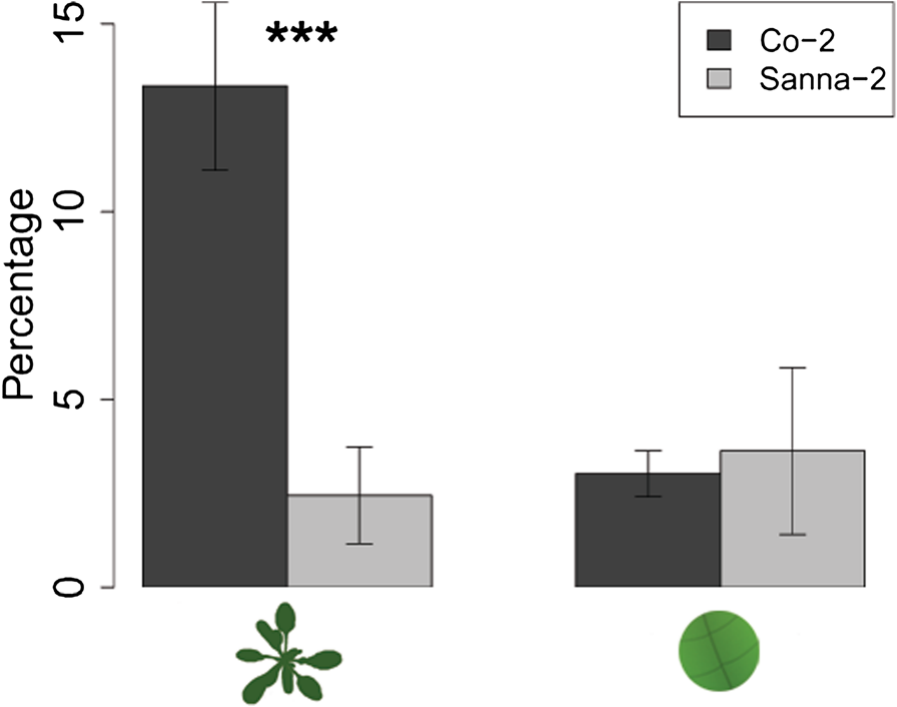
**Contribution of salivation to phloem ingestion.** Percentage of time spent salivating in the phloem compared to the total phloem phase (salivation + ingestion) of *M. persicae* aphids on Arabidopsis accessions Co-2 (resistant) and Sanna-2 (susceptible) (Mann–Whitney U test, *P < 0.05; **P < 0.01; ***P < 0.001, left bars: EPG recording intact plants: n = 19, right bars: EPG recording leaf discs: Co-2 n = 9, Sanna-2 n = 8, error bars represent standard error).

To validate whether automated video tracking delivered a reliable proxy for plant resistance, feeding behaviour of *M. persicae* was measured during 8 hours continuous recording on two natural accessions of Arabidopsis, Co-2 and Sanna-2 (Additional file 3). These accessions were selected from a population of hundreds of accessions based on preliminary video tracking data. Automated video tracking showed that *M. persicae* aphids walked larger distances on Co-2 and reduced the mean duration of long probes (Table 1). EPG recordings on intact plants confirmed shorter durations of (sustained) phloem ingestion, and additionally revealed more short probes, non-probing behaviour and a delayed phloem uptake on Co-2 (Table 1). This behaviour is an indication of both epidermis/mesophyll-located and phloem-located resistance in Co-2 against *M. persicae*. All aphids ingested phloem, but quantitative differences in feeding behaviour between aphids on Co-2 and on Sanna-2 were already apparent in the first hour (Figure 5). A reproduction assay on intact plants confirmed that Co-2 was indeed more resistant than Sanna-2, although the resistance was not absolute. Depending on plant age, aphids either started reproduction later or produced fewer offspring (Figure 6). Although we had been able to correctly identify a quantitative difference in resistance with automated video tracking, the effects were smaller than in EPG recordings on intact plants. To verify whether the plant line effects in the video tracking assay were attenuated due to the use of excised plant tissue, the EPG experiment was repeated with leaf discs. Particularly for the resistant accession, aphid feeding behaviour was different and involved more phloem uptake and fewer short probes on leaf discs compared to intact plants (Additional file 1: Table S2). The only significant difference between the accessions that remained was a reduced duration of phloem uptake events on Co-2 (Table 1). In addition, contribution of salivation to the phloem phase, required to suppress (callose-mediated) sieve-plate occlusion [45], was equal on leaf discs but higher on intact plants of Co-2 (Figure 7). This indicates that the resistance mechanisms in intact plants were partially lost in leaf discs.

**Table 1:** **Feeding behaviour of** ***M. persicae*** **on two Arabidopsis accessions and** ***N. ribisnigri*** **on two lettuce cultivars**

		**Arabidopsis –** ***M. persicae***		**Lettuce –** ***N. ribisnigri***	
		**Co-2 R**	**Sanna-2 S**	**Corbana R**	**Terlana S**
**EPG intact plants**	Total duration non-penetration	139 ± 15	60.2 ± 8.1 Ts***	121 ± 16	29.2 ± 5.8 Tl***
	Total duration phloem feeding	110 ± 13	296 ± 31 M***	2.34 ± 1.68	399 ± 17 M***
	Total duration phloem feeding (>10 min)	27.7 ± 7.9	259 ± 32 M***	0.00 ± 0.00	399 ± 17 M***
	Total duration other penetration activities	231 ± 15.8	124 ± 28 M**	357 ± 16	51.5 ± 14 T***
	Number of non-penetrations	57.7 ± 6.2	22.4 ± 3.1 M***	27.8 ± 7.58	7.58 ± 1.33 T***
	Number of short probes (<3 min)	49.8 ± 5.8	18.4 ± 2.4 M***	13.7 ± 2.0	4.53 ± 1.17 M***
	Number of phloem feeding events	6.37 ± 0.55	4.28 ± 0.70 T*	0.11 ± 0.07	1.16 ± 0.12 M***
	Mean duration of phloem feeding events	17.6 ± 1.4	117 ± 26 M***	22.2 ± 6.16	379 ± 26 M*
	Latency to first phloem feeding event	188 ± 25	111 ± 26 M*	228 ± 94	106 ± 17 M
	Contribution salivation to phloem phase (%)	13.3 ± 2.2	2.45 ± 1.3 M***	98.7 ± 1.0	1.15 ± 0.3 M***
**EPG leaf discs**	Total duration non-penetration	164 ± 48	173 ± 59 M	-	-
	Total duration phloem feeding	220 ± 42	201 ± 48 T	-	-
	Total duration phloem feeding (>10 min)	158 ± 46	177 ± 47 T	-	-
	Total duration other penetration activities	95.3 ± 25.7	106 ± 17 T	-	-
	Number of non-penetrations	19.1 ± 5.7	14.4 ± 3.0 Ts	-	-
	Number of short probes (<3 min)	16.6 ± 4.8	8.63 ± 2.0 Ts	-	-
	Number of phloem feeding events	8.11 ± 1.53	2.63 ± 0.84 M*	-	-
	Mean duration of phloem feeding events	34.2 ± 10.8	99.2 ± 31.7 Ts*	-	-
	Latency to first phloem feeding event	144 ± 51	250 ± 53 T	-	-
	Contribution salivation to phloem phase (%)	3.03 ± 0.6	3.63 ± 2.2 M	-	-
**Video leaf discs**	Total duration non-probing	61.5 ± 9.7	42.6 ± 9.4 T	243 ± 27	148 ± 25 T*
	Total duration long probes (>25 min)	338 ± 20	377 ± 17 T	153 ± 26	283 ± 25 T***
	Total duration sust. probes (>35 min)	276 ± 27	353 ± 19 M*	132 ± 24	260 ± 25 T***
	Total duration other probes (<= 25 min)	80.6 ± 12.4	60.5 ± 9.9 T	84.1 ± 7.0	48.4 ± 9.3 M***
	Number of non-penetrations	33.2 ± 3.9	22.1 ± 3.8 T	31.1 ± 3.1	23.0 ± 2.6 T
	Number of short probes (<3 min)	20.3 ± 3.1	12.4 ± 3.4 T	22.4 ± 3.1	15.6 ± 2.3 M
	Number of long probes (> = 25 min)	5.95 ± 0.36	4.71 ± 0.36 M*	2.19 ± 0.33	3.25 ± 0.30 T*
	Mean duration of long probes (> = 25 min)	62.8 ± 7.0	90.0 ± 9.8 M*	72.4 ± 9.5	99.8 ± 11.2 M*
	Latency to first long probe (> = 25 min)	18.5 ± 5.8	27.1 ± 7.1 M	142 ± 27	65.1 ± 14.2 M*
	Total duration not moving (min)	445 ± 7.3	465 ± 3.5 M	257 ± 27	350 ± 25 T*
	Total distance moved (cm)	46.9 ± 4.6	34.0 ± 4.6 T*	204 ± 47	103 ± 23 T
	Max velocity (mm/s)	0.72 ± 0.12	0.53 ± 0.05 T	2.78 ± 0.54	1.88 ± 0.20 M

Within each EPG and video tracking experiment it was tested whether aphid behaviour differed between the two plant lines. The mean duration and latency were only calculated if the corresponding events did occur (all samples of *M. persicae*; *N. ribsinigri*: EPG n = 2 Corbana and n = 20 Terlana, video tracking n = 21 Corbana and n = 26 Terlana).

Means ± standard error, *P < 0.05; **P < 0.01; ***P < 0.001, T = Student’s *t*-test, Ts = Student’s t-test on square root transformed data, Tl = Student’s t-test on log transformed data, M = Mann–Whitney *U* test, R = resistant plant line, S = susceptible plant line, time is represented in min.

### Benchmarking against EPG recording with lettuce

**Figure 8:**
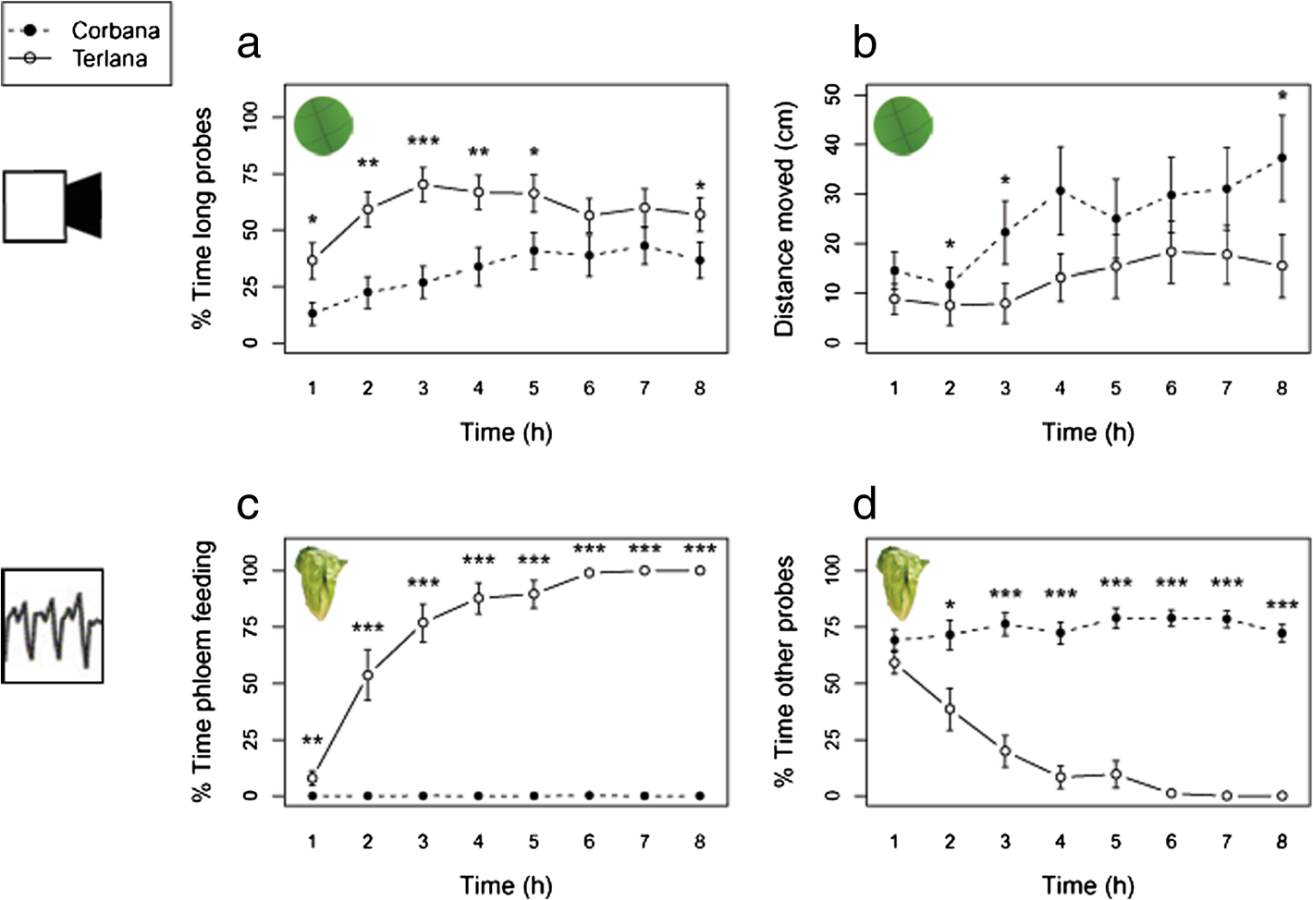
**Behavioural parameters of** ***N. ribisnigri*** **on two lettuce cultivars, Corbana (resistant) and Terlana (susceptible). (a)** Percentage of the time spent on long probes (>25 min), and **(b)** distance moved (cm) were measured by automated video tracking on leaf discs. **(c)** Percentage of the time spent on phloem feeding (waveform 5), and **(d)** percentage of time spent on other probes (pathway, phloem salivation and xylem feeding) were measured by EPGs on intact plants (Mann–Whitney U test per time bin, *P < 0.05; **P < 0.01; ***P < 0.001, video tracking: Corbana n = 27, Terlana n = 28, EPG recording: n = 19).

Apart from a study system with partial resistance, an example of near-complete resistance was tested with the video tracking platform. The behaviour of black-currant lettuce aphids, *N. ribisnigri*, biotype Nr:0 was recorded on two near-isogenic lettuce cultivars, the resistant ‘Corbana’ and susceptible ‘Terlana’. Previous studies showed that the *Nr* gene is responsible for near-complete resistance in Corbana against this biotype of aphids, mainly due to a phloem-located mechanism [34,46]. Our video tracking observations on leaf discs were compared to EPG recording data by ten Broeke et al. [47]. Seven out of nine video tracking variables confirmed that cultivar Corbana was more resistant than cultivar Terlana (Table 1). Aphids on Corbana spent less time on long probes and more time on shorter probes and other activities. In addition, aphids increased their walking activity over time on both cultivars, but generally covered larger distances on Corbana leaf discs (mixed linear model: time effect: P = 0.00, cultivar effect: P = 0.03, time × cultivar interaction: P = 0.77, Figure 8). Yet, the resistance effect was less pronounced in video tracking compared to EPG recording on intact plants: only 11% of the aphids in EPG recordings showed phloem ingestion on Corbana plants, while 78% of the aphids in the video assay performed long probes on Corbana. These long probing events could include other activities, such as water ingestion from xylem vessels, since EPGs showed that on Corbana plants more aphids penetrated xylem sieve elements (12 aphids on Corbana versus 2 aphids on Terlana).

### Throughput

Using simulated data with a similar plant line effect as the data sets from the plant-aphid assays described here, we assessed the required sample size and recording duration for automated video tracking (Table 2). With 20 replicates of 8-hour observations, significant resistance was detected in more than 80% of the cases for the Arabidopsis plant line effect on *M. persicae* (2 response variables tested per simulated data set, Bonferroni correction: P < 0.025). The near-complete resistance of Corbana lettuce against *N. ribisnigri* biotype Nr:0 was detected in more than 80% of the cases with 15 replicates of 4 hours of video observations. Subtle differences in resistance in Arabidopsis were more difficult to detect when video observations were shorter than 8 hours (Table 2). On the other hand, reducing the video duration to the first 4 hours improved the detection of near-complete resistance, as with *N. ribisnigri* biotype Nr:0 on the Corbana lettuce cultivar. Apparently, in this case, the precision of video tracking decreased over time. While the EPG recording with lettuce did not reveal an increase in aphid activities in the xylem or mesophyll over time (Figure 8), the last stretch of the video observation was likely confounded by sessile behaviour other than probing. The risk of falsely rejecting the null hypothesis was limited to 1% (*M. persicae* on Arabidopsis accession Col-0). Overall, video tracking required similar observation durations as EPG recording, but a larger sample size to detect significant plant effects (Table 3). The required amount of replicates was, however, compensated by screening many samples simultaneously and automated data annotation.

**Table 2:** **Required video duration and number of replicates for identifying a significant effect**

**Plant-aphid system**	**Duration**	**Replicates**	**Detection rate**
Arabidopsis - *M. persicae*	8 h	20	> 80%
	6 h	25	> 80%
Lettuce – *N. ribisnigri*	8 h	20	> 80%
	4 h	15	> 80%

Student’s t-tests have been applied to subsamples of two simulated data sets with a similar mean and standard deviation as two response variables from the video tracking assays. The percentage of tests with a significant outcome in at least one of the two variables is represented as the detection rate (Bonferroni correction: P < 0.025).

**Table 3:** **Comparison of automated video tracking and EPG recording characteristics**

	**EPG**	**Video**
Plant material	Intact plants	Leaf discs
Min. observation duration	4-8 h	4-8 h
Min. number of replicates (identification rate ±80%)	3-5	15-20
Maximum sample size per set up	± 8	± 100
Preparation time per sample	± 5 min	± 2 min
Annotation of electrical patterns/video images	± 15 min	Automated

Minimum duration of observations and minimum sample sizes were estimated with simulations. Preparation time per replicate reflects a rough estimation of time required for an experienced person to prepare one arena, resp. plant-aphid individual, for a recording.

## Discussion

### Leaf discs

The effect sizes measured in video tracking with leaf discs were substantially smaller compared to EPG recording on intact plants. EPG recording on leaf discs confirmed that the application of excised plant tissue partially impaired plant resistance [47,48], possibly due to the interrupted supply of ions and metabolites in the phloem, or due to the interference by jasmonic acid and ethylene mediated wound responses [49]. Furthermore, aphids can be disturbed by the decrease in pressure in the sieve elements of excised plant tissue, although they are well capable of active uptake of sap [50,51]. The increase of coagulating proteins and cellular debris in the phloem after plant wounding may plug sieve plates and the aphid’s food canal in the stylets [28,52,53]. To prevent such potential clogging of sieve elements, aphids might increase the injection of watery saliva into the phloem or shorten their feeding events, but neither of these effects were observed consistently. To maintain turgor better the use of leaves still connected to intact plants would be favourable, but this is currently not feasible in view of poor detection of aphids in more complex environments. Arenas designed to hold entire detached leaves or seedlings on agar could, however, be a feasible alternative to leaf discs.

### Application

High-throughput phenotyping techniques of sucking insect species are urgently needed in view of functional genomics studies aiming to find subtle allelic differences in plant populations measuring many hundreds of plants. Conventional methods, like EPG and population studies, are less scalable for this purpose and carry much higher investments in terms of time (labour, duration) and costs (equipment, greenhouses). In this study, automated video tracking was used to study aphid feeding behaviour, but it could as well be applied to track the behaviour of other piercing-sucking insects. We recommend to validate the velocity thresholds for each species first, by checking several video files by hand. As shown here with two aphid species, size and velocity can differ and will affect the accuracy of probe estimations. When studying plants with thick or dark leaves, increased resolution, better (macro) lenses, and lateral light sources instead of backlight can help to improve the detection of insects. We worked with EthoVision XT video tracking and analysis software, but other programs or programming environments, such as MatLab and ImageJ, could as well serve as robust video tracking tools [37,40,54].

## Conclusions

The aim of this study was to develop a high-throughput method to screen large plant populations for resistance to aphids and other piercing-sucking insects. For the first time it is shown that automated video tracking of aphid body movement can be used to estimate how often the insects are penetrating plant tissue and are reaching the vascular bundle. The use of leaf discs instead of intact plants enhanced the throughput of the video tracking platform, but EPG recording illustrated that resistance effects were partially lost in leaf discs. Nevertheless, we could identify both intermediate and extreme levels of resistance with video tracking. In Arabidopsis accession Co-2, we found a quantitative resistance level. This was confirmed in additional bioassays, suggesting the involvement of constitutive or rapidly activated resistance mechanisms in both epidermis/mesophyll and phloem, resulting in a small detrimental effect on the aphid population. The video tracking platform also confirmed the near-complete resistance of the lettuce cultivar Corbana to *N. ribisnigri* biotype Nr:0. Although video tracking requires more replicates to identify resistant plants than the conventional EPG technique, it can screen many samples simultaneously in a confined space. In addition, computerized data acquisition reduces laborious exercises, such as annotation of electrical patterns or counting of aphid populations, and only little plant material is required which can be advantageous when studying segregating populations with only one plant per genotype. These features make automated video tracking a valuable phenotyping method for screening large plant populations for resistance to piercing-sucking insects that are serious pests in our crops.

## Methods

### Plants and insects

Arabidopsis, *Arabidopsis thaliana* (L.) Heynh., plants were grown for 4–5 weeks in pots (5 cm diameter) with pasteurized potting soil (4 h at 80°C; Lentse potgrond, Lent, The Netherlands) in a climate room at 21 ± 1°C, 50-70% relative humidity, an 8/16 h day/night cycle, and a light intensity of 200 μmol m^−2^ s^−1^. Four natural accessions of Arabidopsis were used throughout this study: Col-0 (CS76113), Van-0 (CS76297), Co-2 (CS28163) and Sanna-2 (CS76223). Seeds were acquired from the European Arabidopsis Stock Centre and propagated by the Laboratory of Genetics, Wageningen University.

Lettuce, *Lactuca sativa* (L.), cultivars Corbana (resistant) and Terlana (susceptible) were grown for 3 to 4 weeks in a greenhouse compartment at a temperature of 20 ± 3°C during the day and 18 ± 3°C during the night, 50-70% relative humidity and a 14/10 h day/night cycle using artificial lighting. Seeds were acquired from Enza Zaden bv. *Myzus persicae* (Sulzer) aphids were reared in a climate room on radish plants at 19°C, 50-70% relative humidity and a 16/8 h day/night cycle. *Nasonovia ribisnigri* (Mosely) biotype Nr:0 aphids were reared on the susceptible lettuce cultivar Terlana in a greenhouse compartment at a temperature of 20 ± 3°C during the day and 18 ± 3°C during the night, 50-70% relative humidity, and a 14/10 h day/night cycle.

### Video tracking platform

Aphid behaviour was recorded with an analogue, monochrome camera (Ikegami, model: I CD-49E, type: REV, 768 × 576 pixels) with a varifocal lens (Computar H3Z4512 CS-IR, 4.5-12.5 mm F1.2) mounted above the arenas (Figure 1). An arena consisted of a well in a 96-well microtitre plate, having a 6.5 mm inner diameter (Sarstedt, sterile flat bottom suspension cells. No. 831835500), containing a leaf disc with the abaxial side up on a substrate of 1% agar (technical agar no.3, Oxoid). One aphid was introduced per arena and cling film was tightly wrapped around the plate to prevent aphids from escaping. The microtitre plate was placed on a platform, 1 cm above a backlight unit (FL tubes, 5000 K). A fan was attached between the platform and backlight unit to prevent water condensation inside the arenas. Room temperature was controlled at 21-22°C.

### Software settings

EthoVision XT 8.5 video tracking and analysis software (Noldus Information Technology bv, Wageningen, The Netherlands) was used for automated video tracking of aphid feeding behaviour in multiple arenas simultaneously [41,55]. Subject detection was achieved with grey scaling (Additional file 1: Table S1). Arenas contained two zones: the leaf disc (zone 1) and the space surrounding the leaf disc (zone 2) (Additional file 1: Figure S1). Zone 1 had a diameter of approximately 5 mm, excluding the outer edges of the leaf disc to prevent aphids on the arena wall to be falsely assigned to the leaf disc. Because zone 1 and zone 2 required different grey scale thresholds, optimal thresholds for zone 1, the leaf disc, were chosen. Consequently, only behavioural data acquired in zone 1 were used throughout this study. Velocity and time thresholds appropriate to starting and ending a probe were fine-tuned using simultaneous observations of the top-view camera (275 pixels per mm^2^) and a side-view camera attached to a stereo microscope (20-40 × magnification), capturing close-up recording of proboscis and antennae movements of *M. persicae* aphids (Additional file 2). A probe start was automatically recorded when the aphid was positioned on the leaf disc and its velocity dropped below 0.02 mm/s and did not exceed 0.3 mm/s for at least 10 seconds (Figure 2, Additional file 1: Figure S2). A probe stop was recorded when aphid velocity exceeded 0.3 mm/s for the relatively small wingless *M. persicae* or 0.35 mm/s for the larger winged *N. ribisnigri* and did not decrease below 0.1 mm/s for at least 2 seconds. Confounding movements during probing were generally characterized by a repetitive pattern of short movements. The 2 seconds time delay prevented that these movements resulted in false probe stops. Zone-transition problems, which occurred when aphids were positioned exactly on the edge of zone 1 and zone 2, were filtered from the data set after acquisition in EthoVision XT, with the statistical computing program R (Additional file 4). These incidences, characterized by a train of consecutive short probes in the output, were filtered out by excluding probes with a duration of less than 3 seconds that were preceded by a very short non-probe bout of maximally 15 seconds. These thresholds were selected by hand using some examples of zone transition problems in this study.

### Video recording versus human observations

To validate automated tracking of probes with manual scoring, we used a camera mounted on a stereo microscope (20-40×) with a side-view on a single arena (n = 16) (Additional file 2). Each replicate consisted of a 55 min continuous recording of one arena with a single adult *M. persicae* aphid and an Arabidopsis leaf disc, by both the top-view and side-view camera. Aphids were starved between 30 minutes and three hours before the experiment. Recordings with the top-view camera were performed at two distances: capturing 20 arenas with 275 pixels per mm^2^, and capturing 35 arenas with 155 pixels per mm^2^. Leaf discs of 6 mm in diameter were cut just below the leaf apex of 4–5 week old Col-0 and Van-0 plants. The Observer® XT 10 software (Noldus Information Technology bv, Wageningen, The Netherlands) was used for manual scoring of probes. Probe starts were manually recorded when body movement stopped, the proboscis was touching the leaf and antennae moved backwards. If the aphid’s proboscis was obscured, body arrestment on the leaf disc with subsequent backward movement of antennae was defined as a probe start [56,57]. Probe endings were manually recorded when antennae moved upward and the aphid removed its proboscis from the leaf, or, when the latter was not visible, when the antennae moved upwards followed by locomotion. Apart from probe estimations, we also tracked the “total time not moving” across the whole observation, using a start velocity of 0.3 mm/s and a stop velocity of 0.02 mm/s. Velocities were averaged across 5 frames, using a sample rate of 5 frames per second.

### Video tracking assays

In each recording twenty arenas were tracked simultaneously for 8 hours, with a frame rate of 25 s^−1^, and a resolution of 275 pixels per mm^2^ (Additional file 3). All arenas consisted of a different plant and aphid individual and within each recording the 2 involved plant lines were equally represented. For Arabidopsis accessions Co-2 and Sanna-2, automated video tracking was performed with 7 to 8 day old wingless *M. persicae* aphids (Co-2 n = 20, Sanna-2 n = 17). Leaf discs of 6 mm in diameter were made just below the apex of intermediately aged leaves. Aphid survival was checked the day after recording. Subject detection was checked after data acquisition on 6 time points across the video. Three samples with no or low quality detection were excluded from the analysis. Video tracking of winged *N. ribisnigri* biotype Nr:0 on lettuce cultivars Terlana and Corbana was performed with 4 mm leaf discs (Corbana n = 27, Terlana n = 28). In view of the large contour of winged *N. ribisnigri* aphids, we used arenas with leaf discs of 4 mm diameter and a 3–4 mm leaf edge-to-wall distance in order to have a clear distinction between aphids on the leaf disc and aphids on agar or the arena wall. Leaf discs were made near the leaf base of the third oldest leaf, next to the mid vein. None of the aphids had died the day after recording. Five samples with no or low quality detection were excluded from the analysis. The response variable “duration not moving” was measured using a start velocity of 0.3 mm/s and a stop velocity of 0.02 mm/s. Velocities were averaged across 5 frames, using a sample rate of 5 frames per second.

### EPG recording

Feeding behaviour of the green peach aphid, *M. persicae*, was analysed with EPG recording on two natural accessions of Arabidopsis, Co-2 and Sanna-2, during 8-hour observations. EPG recording was made on both intact plants (Co-2 n = 19, Sanna-2 n = 18) and leaf discs (Co-2 n = 9, Sanna-2 n = 8), using direct currents (DC) according to the methodology of ten Broeke et al. [34]. An electrode was inserted in the potting soil or agar respectively, and a thin gold wire (1.5 cm length for intact plants, 1 cm length for leaf discs) was gently attached to the dorsum of 8 to 11 day old wingless aphids with silver glue. The electrical circuit was completed when the aphid’s piercing-sucking mouthparts penetrated the plant cuticle and the electrical signals, correlated to stylet activities, were recorded instantly [25]. Each replicate consisted of a different aphid and plant individual, employing one leaf disc per plant. Leaf discs of 9 mm in diameter were processed just below the apex of intermediately aged Arabidopsis leaves and placed abaxial side up in a Petri dish on a 1% agar substrate. A transparent plastic sheet covered the agar surrounding the leaf disc to prevent aphids to get stuck or make probes in the agar. Aphids that did not start probing within the first 3 hours of the observation were excluded from the analysis. EPG recording of winged *N. ribisnigri* biotype Nr:0 on lettuce cultivars Corbana and Terlana has been made in a previous study by ten Broeke et al. [47] (8-hour recording, n = 19).

### Aphid population development

One *M. persicae* neonate (0 to 24 h old) was transferred to each Arabidopsis plant in a climate chamber (21 ± 1°C, 50-70% relative humidity, an 8/16 h day/night cycle, light intensity of 200 μmol m^−2^ s^−1^). In the first assay 2.5-week-old plants were infested, in the second assay 3.5-week-old plants. A soap-diluted water barrier prevented aphids from moving between plants. Six, seven, and eight days after introduction the presence of the aphid and its offspring was checked. None of the aphids developed wings. 14 Days after infestation the number of aphids was counted per plant. Plants without an adult aphid 8 days after introduction, and plants without any adults or neonates 14 days after introduction were excluded from the analysis (assay 1: Co-2 n = 19, Sanna-2 n = 15; assay 2: Co-2 n = 14, Sanna-2 n = 13).

### Simulations

In simulations, 10^4^ random draws were taken from a normal distribution with the mean and standard deviation of a response variable of the Arabidopsis-*M. persicae* and lettuce-*N. ribisnigri* data sets (Additional file 1: Table S3). For video observations data was simulated with two probing variables: the mean duration of long probes and the total duration of sustained probes. For EPGs the total duration of phloem ingestion was simulated. Random draws were excluded when values were below zero, below the minimum duration of the probe category, or above the maximum recording duration. The generated data sets were subsampled with 1000 iterations without replacement for several replicate levels (n = 10, 15, 20, 25, 30, 35, 40). Student’s t-tests were executed for each iteration and the percentage of significant p-values per replicate level was calculated. Video tracking simulation tests were defined significant if they had a P-value below α = 0.025 for at least one of the two probing variables (Bonferroni correction: α = 0.05/2). For EPG simulations one variable and P-values below α = 0.05 already delivered maximum detection rates. This process was performed on complete data sets of EPG and video recording (8 h observations) and on data sets rescaled to shorter durations (6 and 4 hour observations). The proportion of tests where the null hypothesis is incorrectly rejected, was calculated with simulations based on a data set of 8-hour video recording of *M. persicae* on Arabidopsis accession Col-0 (data set n = 53, replicate levels n = 15 and n = 20, two variables, P < 0.025, Additional file 1: Table S3).

### Statistical analysis

An R script was written to calculate response variables of video tracking, such as the total number and total duration of short and long probes in each observation and for each hour (Additional file 4). For EPG recording, the start time and duration of waveforms were analysed with the EPG PROBE 3.0 software (EPG-Systems, Wageningen, The Netherlands). Further calculations and analyses of EPG data were performed with the statistical computing program R. The duration of phloem ingestion events in EPG recording were calculated as the sum of three subsequent waveforms: (a) inter- and intracellular penetrations followed by (b) phloem salivation and (c) phloem ingestion. Bar graphs were produced with the R package sciplot version 1.1-0 (Morales 2012) [58]. Data distributions and homogeneity of variances were tested with a Shapiro test and a Levene’s test. In case data transformations (square root, log, logit, arcsine) did not result in a distribution that approaches a normal distribution, non-parametric tests were applied. Human observations were compared to video tracking parameters with a paired *t*-test or, when data were not normally distributed with a Wilcoxon signed ranks matched pairs test. Correlations were tested with a Pearson correlation test or, when data were not normally distributed, with a Spearman correlation test. For benchmarking of video tracking against EPGs with susceptible and resistant Arabidopsis and lettuce lines and for the reproduction assay, response variables were tested with a Student’s *t*-test, or when the data were not normally distributed with a Mann–Whitney *U* test. Walking activity of aphids was tested across 8 time bins of 1 hour. The distance moved was not normally distributed and, therefore, transformed to ranks ranging from the lowest to highest value within the complete data set. A mixed linear model was applied on the ranks, using plant line, time bin, and plant line x time bin interaction as fixed effects and plant/aphid individual as a random effect.

## Additional files

Additional file 1: Figure S1. Arena settings. **Figure S2.** Trial Control Settings. **Table S1.** Subject detection settings for *M. persicae*. **Table S2.** Absolute differences between EPG recording and automated video tracking. **Table S3.** Video tracking variables used to generate simulations. Additional file 2:
**Movie of aphid feeding behaviour measured by manual annotations and automated video tracking simultaneously.** Events were estimated where the *M. persicae* aphid penetrated the Arabidopsis leaf disc with its piercing-sucking mouth parts. Manual scoring of probes was achieved with a side-view camera mounted on a stereo microscope and The Observer XT software. Automated video tracking was performed with a top view on the arena (275 pixels/mm) and EthoVision XT software (Noldus Information Technology bv, Wageningen, The Netherlands). EthoVision XT recorded a probe when the aphid’s centre point was located on the leaf disc and did not move. Other motion parameters, such as the distance moved (cm) and velocity (cm/s), were also acquired with automated tracking. The movie is displayed at double speed. Additional file 3:
**Movie of automated video tracking of 20 aphids simultaneously.** *M. persicae* aphids were tracked on Arabidopsis leaf discs using EthoVision XT video tracking software (Noldus Information Technology bv, Wageningen, The Netherlands). The aphid’s centre point (red) was automatically detected and tracks of the previous 30 seconds are visualised in red. Arenas are numbered from 1 (top left) to 20 (bottom right). The movie is displayed at 16× speed. Additional file 4:
**R script for feature extraction from EthoVision XT output.**

## Abbreviation

EPG: Electrical Penetration Graph

## References

[1] Blackman RL, Eastop VF (2006). Aphids on the world’s herbaceous plants and schrubs.

[2] Tjallingii WF, Chapman RF, De Boer G (1995). Regulation of phloem sap feeding by aphids. Regulatory mechanisms in insect feeding.

[3] Minks AK, Harrewijn P (1989). World crop pests. Aphids. Their biology, Natural enemies and control.

[4] De Vos M, Van Oosten VR, Van Poecke RMP, Van Pelt JA, Pozo MJ, Mueller MJ, Buchala AJ, Metraux JP, Van Loon LC, Dicke M, Pieterse CMJ (2005). Signal signature and transcriptome changes of *Arabidopsis* during pathogen and insect attack. MPMI.

[5] Eigenbrode SD, Espelie KE (1995). Effects of plant epicuticular lipids on insect herbivores. Annu Rev Entomol.

[6] Wang E, Wang R, DeParasis J, Loughrin JH, Gan S, Wagner GJ (2001). Suppression of a P450 hydroxylase gene in plant trichome glands enhances natural-product-based aphid resistance. Nat Biotechnol.

[7] Ceci LR, Volpicella M, Rahbé Y, Gallerani R, Beekwilder J, Jongsma MA (2003). Selection by phage display of a variant mustard trypsin inhibitor toxic against aphids. Plant J.

[8] Mewis I, Appel HM, Hom A, Raina R, Schultz JC (2005). Major signaling pathways modulate *Arabidopsis* glucosinolate accumulation and response to both phloem-feeding and chewing insects. Plant Physiol.

[9] Smith CM, Boyko EV (2007). The molecular bases of plant resistance and defense responses to aphid feeding: current status. Entomol Exp Appl.

[10] Pegadaraju V, Louis J, Singh V, Reese JC, Bautor J, Feys BJ, Cook G, Parker JE, Shah J (2007). Phloem-based resistance to green peach aphid is controlled by Arabidopsis PHYTOALEXIN DEFICIENT4 without its signaling partner ENHANCED DISEASE SUSCEPTIBILITY1. Plant J.

[11] Du Y, Poppy GM, Powell W, Pickett JA, Wadhams LJ, Woodcock CM (1998). Identification of semiochemicals released during aphid feeding that attract parasitoid *Aphidius ervi*. J Chem Ecol.

[12] Will T, van Bel AJE (2006). Physical and chemical interactions between aphids and plants. J Exp Bot.

[13] Kollner TG, Held M, Lenk C, Hiltpold I, Turlings TCJ, Gershenzon J, Degenhardt J (2008). A maize (E)-beta-caryophyllene synthase implicated in indirect defense responses against herbivores is not expressed in most American maize varieties. Plant Cell.

[14] Wink M (1988). Plant breeding: importance of plant secondary metabolites for protection against pathogens and herbivores. Theor Appl Genet.

[15] Eenink AH, Dieleman FL (1977). Screening *Lactuca* for resistance to *Myzus persicae*. Neth J Plant Pathol.

[16] Smyrnioudis IN, Harrington R, Katis NI (2002). A simple test for evaluation of transmission efficiency of barley yellow dwarf virus by aphids. Phytoparasitica.

[17] Chen X, Vosman B, Visser RGF, van der Vlugt RAA, Broekgaarden C (2012). High throughput phenotyping for aphid resistance in large plant collections. Plant Methods.

[18] Stelinski L, Tiwari S (2013). Vertical T-maze choice assay for arthropod response to odorants. J Vis Exp.

[19] Kloth KJ, Thoen MPM, Bouwmeester HJ, Jongsma MA, Dicke M (2012). Association mapping of plant resistance to insects. Trends Plant Sci.

[20] Smith CM, Khan ZR, Pathak MD (1994). Techniques for evaluating insect resistance in crop plants.

[21] Broekgaarden C, Poelman EH, Steenhuis G, Voorrips RE, Dicke M, Vosman B (2008). Responses of *Brassica oleracea* cultivars to infestation by the aphid *Brevicoryne brassicae*: an ecological and molecular approach. Plant Cell Environ.

[22] Mewis I, Tokuhisa JG, Schultz JC, Appel HM, Ulrichs C, Gershenzon J (2006). Gene expression and glucosinolate accumulation in *Arabidopsis thaliana* in response to generalist and specialist herbivores of different feeding guilds and the role of defense signaling pathways. Phytochemistry.

[23] Moran PJ, Thompson GA (2001). Molecular responses to aphid feeding in Arabidopsis in relation to plant defense pathways. Plant Physiol.

[24] McLean DL, Kinsey MG (1964). A technique for electronically recording aphid feeding and salivation. Nature.

[25] Tjallingii WF, Minks AK, Harrewijn P (1988). Electrical recording of stylet penetration activities. Aphids, their biology, natural enemies and control.

[26] van Emden HF, Harrington R (2007). Aphids as crop pests.

[27] van Helden M, Tjallingii WF (1993). Tissue localisation of lettuce resistance to the aphid N. ribisnigri using electrical penetration graphs. Entomol Exp Appl.

[28] Tjallingii WF (2006). Salivary secretions by aphids interacting with proteins of phloem wound responses. J Exp Bot.

[29] Will T, Tjallingii WF, Thönnessen A, Van Bel AJE (2007). Molecular sabotage of plant defense by aphid saliva. Proc Natl Acad Sci U S A.

[30] Boquel S, Giordanengo P, Ameline A (2011). Divergent effects of PVY-infected potato plant on aphids. Eur J Plant Pathol.

[31] Nalam VJ, Keeretaweep J, Sarowar S, Shah J (2012). Root-derived oxylipins promote green peach aphid performance on arabidopsis foliage. Plant Cell.

[32] ten Broeke CJM, Dicke M, van Loon JJA (2013). Resistance in a *Lactuca virosa* accession to a new biotype of *Nasonovia ribisnigri*. Euphytica.

[33] Alvarez AE, Tjallingii WF, Garzo E, Vleeshouwers V, Dicke M, Vosman B (2006). Location of resistance factors in the leaves of potato and wild tuber-bearing *Solanum* species to the aphid *Myzus persicae*. Entomol Exp Appl.

[34] ten Broeke CJM, Dicke M, van Loon JJA (2013). Performance and feeding behaviour of two biotypes of the black currant-lettuce aphid, *Nasonovia ribisnigri*, on resistant and susceptible *Lactuca sativa* near-isogenic lines. Bull Entomol Res.

[35] Bakchine E, Pham-Delegue MH, Kaiser L, Masson C (1990). Brief communication: computer analysis of the exploratory behavior of insects and mites in an olfactometer. Physiol Behav.

[36] Shcherbakov D, Schill RO, Brümmer F, Blum M (2010). Movement behaviour and video tracking of *Milnesium tardigradum* Doyère, 1840 (Eutardigrada, Apochela). Contrib Zool.

[37] Pinkiewicz TH, Purser GJ, Williams RN (2011). A computer vision system to analyse the swimming behaviour of farmed fish in commercial aquaculture facilities: a case study using cage-held atlantic salmon. Aquac Eng.

[38] Moreno-Delafuente A, Garzo E, Moreno A, Fereres A (2013). A plant virus manipulates the behavior of its whitefly vector to enhance its transmission efficiency and spread. PLoS ONE.

[39] Spitzen J, Spoor CW, Grieco F, ter Braak C, Beeuwkes J, van Brugge SP, Kranenbarg S, Noldus LPJJ, van Leeuwen JL, Takken W (2013). A 3D analysis of flight behavior of *Anopheles gambiae* sensu stricto malaria mosquitoes in response to human odor and heat. PLoS ONE.

[40] Dell AI, Bender JA, Branson K, Couzin ID, de Polavieja GG, Noldus LPJJ, Pérez-Escudero A, Perona P, Straw AD, Wikelski M, Brose U (2014). Automated image-based tracking and its application in ecology. Trends Ecol Evol.

[41] Noldus LPJJ, Spink AJ, Tegelenbosch RAJ (2002). Computerised video tracking, movement analysis and behaviour recognition in insects. Comput Electron Agric.

[42] Tjallingii WF (1994). Sieve element acceptance by aphids. Eur J Entomol.

[43] Prado E, Tjallingii WF (2007). Behavioral evidence for local reduction of aphid-induced resistance. J Insect Sci.

[44] Grieco F, Loijens L, Krips OE, Smit G, Spink AJ, Zimmerman P (2010). EthoVision XT reference manual.

[45] Will T, Kornemann SR, Furch ACU, Tjallingii WF, van Bel AJE (2009). Aphid watery saliva counteracts sieve-tube occlusion: a universal phenomenon?. J Exp Biol.

[46] Reinink K, Dieleman FL (1989). Comparison of sources of resistance to leaf aphids in lettuce (lactuca sativa L.). Euphytica.

[47] Ten Broeke CJM (2013). Unravelling the resistance mechanism of lettuce against *Nasonovia ribisnigri*.

[48] Liu Y, McCreight JD (2006). Responses of *Nasonovia ribisnigri* (Homoptera: Aphididae) to susceptible and resistant lettuce. J Econ Entomol.

[49] Leon J, Rojo E, Sanchez-Serrano JJ (2001). Wound signalling in plants. J Exp Bot.

[50] Will T, Hewer A, Van Bel AJE (2008). A novel perfusion system shows that aphid feeding behaviour is altered by decrease of sieve-tube pressure. Entomol Exp Appl.

[51] Mittler TE, Minks AK, Harrewijn P (1988). Applications of artificial feeding techniques for aphids. Aphids, their biology, natural enemies ano control.

[52] Turgeon R (2010). The puzzle of phloem pressure. Plant Physiol.

[53] Martin B, Rahbé Y, Fereres A (2003). Blockage of stylet tips as the mechanism of resistance to virus transmission by *Aphis gossypii* in melon lines bearing the Vat gene. Ann Appl Biol.

[54] Husson SJ, Steuer Costa W, Schmitt C, Gottschalk A, Community eTCeR (2012). Keeping track of worm trackers. Wormbook.

[55] Noldus LPJJ, Spink AJ, Tegelenbosch RAJ (2001). EthoVision: a versatile video tracking system for automation of behavioral experiments. Behav Res Methods Instrum Comput.

[56] Hardie J, Holyoak M, Taylor NJ, Griffiths DC (1992). The combination of electronic monitoring and video-assisted observations of plant penetration by aphids and behavioural effects of polygodial. Entomol Exp Appl.

[57] Hardie J, Powell G, Walker PG, Backus EA (2000). Close-up video combined with electronic monitoring of plant penetration and behavioral effects of an aphid (Homoptera: Aphididae) antifeedant. Principles and applications of electronic monitoring and other techniques in the study of Homopteran feeding behavior.

[58] R-Core-Team (2013). R: a language and environment for statistical computing.

