# Supplementary material for: High-throughput phenotyping of plant resistance to aphids by automated video tracking
Source: Plant Methods. 2015 Jan 30;11:4. doi: 10.1186/s13007-015-0044-z (PMC4318543; doi:10.1186/s13007-015-0044-z)
Supplement: Additional file 1: Figure S1. — Arena settings. Figure S2. Trial Control Settings. Table S1. Subject detection settings for M. persicae. Table S2. Absolute differences between EPG recording and automated video tracking. Table S3. Video tracking variables used to generate simulations. [file 13007_2015_44_MOESM1_ESM.pdf]

Additional file 1

High-throughput phenotyping of plant resistance to aphids by automated video tracking

Karen J. Kloth[1,2,3], Cindy J.M. ten Broeke[1], Manus P.M. Thoen[1,2,3], Marianne Hanhart-van den Brink[1], Gerrie L. Wiegers[1,3], Olga E. Krips[4], Lucas P.J.J. Noldus[4], Marcel Dicke[1] and Maarten A. Jongsma[3]

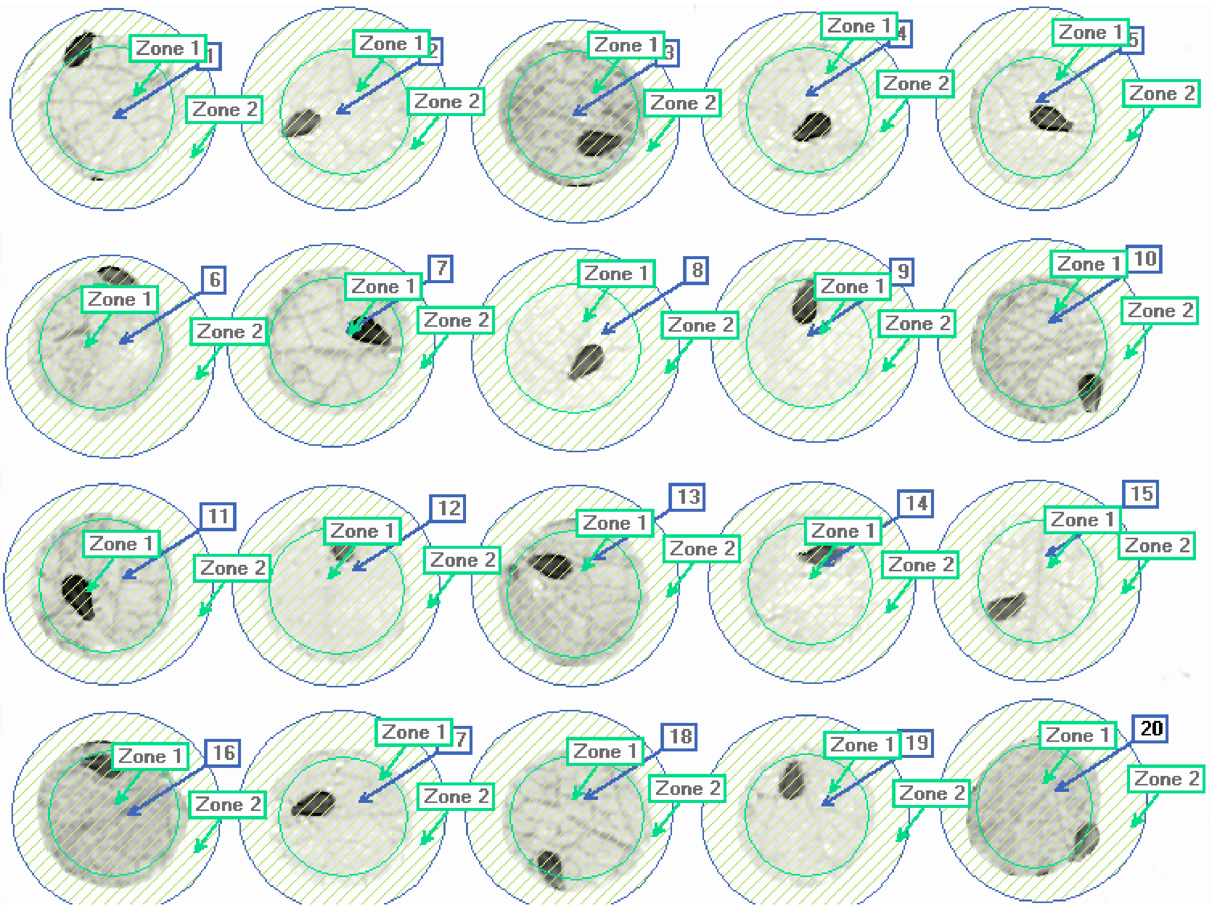

Figure S1 Arena settings in EthoVision XT.

Trial and control settings

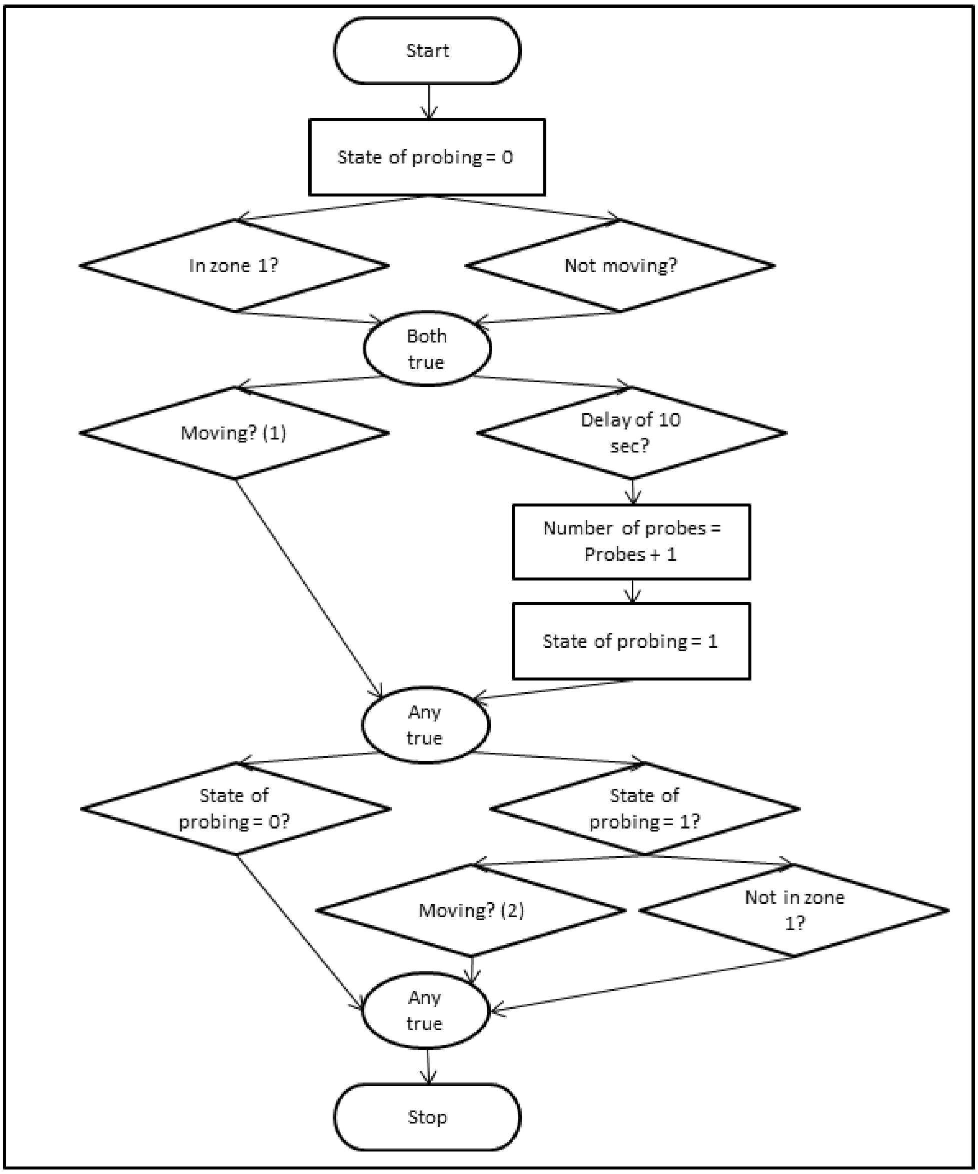

Figure S2 Trial Control Settings in EthoVision XT for measuring the start time and duration of probes. Zone 1 is the leaf disc. ‘Not moving’ and ‘Moving (1)’ were defined by a start velocity of 0.3 mm/s and a stop velocity of 0.02 mm/s, averaged over 5 samples (0.2 seconds). The condition ‘Moving (2)’ was defined by a start velocity of 0.3 mm/s for *M. persicae*, resp. 0.35 mm/s for *N. ribsinigri*, a stop velocity of 0.1 mm/s, and a minimum duration of 2 seconds. This procedure was repeated indefinitely during data acquisition. Velocities were calculated by taking the average velocity of 5 frames, with a frame rate of 5 frames per second.

Table S1 Subject detection settings in EthoVision XT for *M. persicae*.

**Parameters**	**Settings**
Detection method	Grey scaling, centre point detection
Grey scale range	6-200
Subject size	85-600 pixels
Contour erosion (first)	2 pixels
Contour dilation	2 pixels
Sample rate	5 samples per second
Pixel smoothing	Medium
Track noise reduction	Off
Track smoothing profile	Lowess, 10 samples before and after

Table S2 Absolute differences between EPG recording and automated video tracking. Response variables are compared between (1) EPGs on intact plants, (2) EPGs on leaf discs, and (3) automated video tracking. Related variables, such as duration of phloem ingestion in EPGs and duration of long probes in video tracking, were tested for absolute differences (Kruskal-Wallis test, pairwise comparison with Mann-Whitney U test, alpha=0.05). On Arabidopsis accessions Co-2 and Sanna-2, probing behaviour of *M. persicae* was recorded, on lettuce cultivars Corbana and Terlana, probing behaviour of *N. ribisnigri*. Within each plant-aphid combination, different colours represent significant differences between the screening methods. Dark colours represent significantly higher values than lighter colours. Blocks with a cross were not significantly different from other blocks within the plant-aphid combination.

	Co-2			Sanna-2			Corbana		Terlana	
	EPG intact plant	EPG leaf disc	Video leaf disc	EPG intact plant	EPG leaf disc	Video leaf disc	EPG intact plant	Video leaf disc	EPG intact plant	Video leaf disc
Total duration not penetrating/not probing										
Total duration phloem feeding /long probes										
Total duration sustained phloem feeding/long probes										
Total duration other penetrations/other probes										
Number of non-penetrations										
Number of phloem feeding events/long probes										
Number of short probes										
Mean duration phloem feeding events/long probes										
Latency to phloem feeding events/long probes										

Table S3 Video tracking variables used to generate simulations.

Plant - aphid	Duration	Trait (min)	R	S
Arabidopsis - *Myzus*	8h	Mean duration long probes	62.8 ± 31.1	90.0 ± 40.4
		Total duration sustained probes	276 ± 120	353 ± 77.0
Arabidopsis - *Myzus*	6h	Mean duration long probes	62.6 ± 31.9	84.9 ± 32.0
		Total duration sustained probes	208 ± 91.9	263 ± 62.5
Lettuce - *Nasonovia*	8h	Mean duration long probes	72.4 ± 43.6	99.8 ± 57.3
		Total duration sustained probes	132 ± 127	260 ± 133
Lettuce - *Nasonovia*	4h	Mean duration long probes	53.0 ± 29.9	87.9 ± 49.4
		Total duration sustained probes	44.1 ± 60.8	125 ± 77.2

Plant - aphid	Duration	Trait (min)	Col-0
Arabidopsis - *Myzus*	8h	Mean duration long probes	55.6 ± 30.9
		Total duration sustained probes	170 ± 110

Mean ± standard deviation, R= resistant plant line (Co-2, resp. Corbana), S= susceptible plant line (Sanna-2, resp. Terlana).
